# Myocardial Function in Offspring Aged 5 to 8 Years of Pregnancy Complicated by Severe Preeclampsia Measured by Two-Dimensional Speckle-Tracking Echocardiography

**DOI:** 10.3389/fphys.2021.643926

**Published:** 2022-01-06

**Authors:** Huiyun Chen, Yu Gong, Fangcan Sun, Bing Han, Bingyuan Zhou, Jiali Fan, Xinxian Gu

**Affiliations:** ^1^The First Affiliated Hospital of Soochow University, Suzhou, China; ^2^Suining Central Hospital, Suining, China; ^3^Dushu Lake Hospital Affiliated to Soochow University, Suzhou, China

**Keywords:** myocardial strain, myocardial function, premature birth (MeSH), heart rate (HR), preeclampsia

## Abstract

**Objective:** This study aimed to quantitatively assess myocardial strain in preterm children aged 5 to 8 years of pregnancy complicated by severe preeclampsia (PE) by two-dimensional (2D) speckle tracking echocardiography.

**Method:** A cohort study of 23 preterm children delivered by severe PE pregnant women from 2010 to 2012 in the First Affiliated Hospital of Soochow University was carried out. 23 preterm children from uneventful pregnancies in the same period served as controls. Myocardial functions including left ventricular longitudinal strain, radial strain, circumferential strain, and right ventricular longitudinal strain were evaluated by conventional Doppler, tissue Doppler imaging, and 2D speckle-tracking echocardiography (2D STE). All examinations were performed by an experienced ultrasonographer using the VIVID E9 (GE Healthcare) machine, according to standard techniques.

**Results:** Children aged 5–8 years delivered from severe PE presented less weight (24.41 vs. 20.89 kg, *P* < 0.05), shorter height (124.1 vs 115.6 cm, *P* < 0.05) and faster heart rates (84 vs. 93 bpm, *P* < 0.05) compared to offspring of normotensive women. There were no significant differences in global left ventricular longitudinal strain, radial strain, circumferential strain, and right ventricular longitudinal strain between the children in the experimental group and the control group (*P* > 0.05).

**Conclusion:** Exposure to the intrauterine environment of severe PE during the fetal period did not have a significant impact on cardiac structure in premature children at 5–8 years old, but they had a higher resting heart rate which may be associated with cardiovascular disease in the long run.

## Introduction

Preeclampsia is a common complication during pregnancy. There is evidence that preeclampsia (PE) could cause epigenetic alterations, among which alterations in imprinted genes could affect the process of embryo formation, trophoblastic growth, embryonic and organismal development (Peixoto et al., 2018). Alterations in gene expression or protein expression do not lead to changes in DNA sequence, so it could be stably inherited as cells divide and proliferate. It has also been reported that oxidative stress induced by PE amplifies the systemic inflammatory response, increases the release of misfolded and aggregated proteins and autoantibodies (Cheng and Sharma, 2016). Furthermore, the development of trophoblast invasion and vascular dysplasia could result in placental insufficiency, reduced oxygen and nutrient supply, and destruction of cardiomyocyte growth and myocardial fiber structure. In addition, if coupled with chorionic villi dysplasia or thrombosis, placental resistance, and cardiac afterload would increase. In order to maintain cardiac output, the developing myocardial tissues would undergo macroscopic and microscopic changes in structure and function, leading to myocardial remodeling and dysfunction during the growth and development of offspring (Zhang, 2005; Jonker et al., 2010).

The current studies on the effects of preeclampsia on myocardial function in offspring are inconsistent. Some researchers believe that the damaged cardiac structures gradually recover as the offspring grow and develop. A longitudinal cohort study consisting of 784 Finnish adults (34–49 years) found that slight changes of cardiac structure in adults born small for gestational age (SGA) were unlikely to have pathologic effects on cardiovascular disease (Arnott et al., 2015). However, Drude argued that children born with preeclampsia could lead to an increased late left diastole velocity at mitral valve attachments and a faster heart rate (Fugelseth et al., 2011), suggesting that cardiovascular disease risk persists in the offspring of hypertensive pregnancies, which was in line with a 20-year prospective follow-up birth cohort in Australia (Davis et al., 2015).

The two-dimensional (2D) ultrasound image of the heart is composed of many acoustic spots evenly distributed in the myocardium, which move synchronously with the myocardial tissue. The speckle tracking imaging can continuously track the trajectory of each spot and quantitatively analyze movement speed and strain of the myocardial tissue, which can accurately reflect myocardial function. In the current study, we performed 2D-STD to evaluate cardiac structure and function to explore whether exposure to the intrauterine environment of severe PE during the fetal period caused cardiovascular disease risk in premature children at 5–8 years old and provide a basis for cardiac screening in children.

## Study Populations and the Study Protocol

### Study Populations

The study included 23 preterm children delivered by severe PE pregnant women and 23 age-matched children from uneventful pregnancies from the First Affiliated Hospital of Soochow University.

Severe preeclampsia was diagnosed according to the 2013 edition of the American College of Obstetricians and Gynecologists (ACOG) guidelines when at least one of the following was met: ➀ systolic blood pressure ≥ 160 mmHg and/or diastolic blood pressure ≥ 110 mmHg (on two occasions at least 4 h apart while the patient is on bed rest); ➁ thrombocytopenia (platelet count < 100^∗^109/L); ➂ impaired liver function (as indicated by abnormally elevated blood concentrations of liver enzymes to twice normal concentration); ➃ renal insufficiency (elevated blood creatinine > 97.2 μmol/L or more than twice the normal reference value); ➄ pulmonary edema; ➅ new onset of cerebral or visual disturbances. Patients were excluded if pregnancies were complicated with diseases that may affect the development of fetal heart, such as gestational diabetes mellitus, maternal hematologic diseases, immune system diseases, malignant tumors, heart disease, intrauterine infections, fetal congenital heart disease, or other deformities.

### Transthoracic 2D Datum Acquisition

The 2D echocardiographic imaging was performed by GE Vivid E9 ultrasound scanner with phased array transducers M5S-D automated software (GE Healthcare, Milwaukee, WI, United States) 2–4 MHz transducer. The patient was examined in the left lateral decubitus position and connected to ECG using a Vivid E9 ultrasound scanner with phased array transducers M5S-D. We acquired a 2D datum from the parasternal long-axis and short-axis views and three standard apical views and captured three consecutive cardiac cycles for each view. Pulsed Doppler images were acquired in the apical four-chamber view with the sample volume placed in the orifice of the right upper pulmonary vein and the mitral valve inflow at the leaflet tip. The cross-valve pressure difference was measured in continuous Doppler mode. Tissue-Doppler Images (TDI) analysis was performed to record mitral annulus velocities at septal and lateral walls in the apical four-chamber view.

### Analysis of 2D and Doppler Parameters

According to the recommendations of the American Society of Echocardiography, aortic root diameter left atrial diameter, interventricular septum, LVES D, and LVED D were measured from M-mode echocardiograms. The peak early (E) and late (A) trans-mitral flow velocities, the ratio of early-to-late peak velocities (E/A), and mitral flow deceleration time (DT) were measured from the mitral Doppler flow waveform. Pulsed-wave Doppler velocities of the pulmonary venous flow were obtained to analyze pulmonary vein inflow for peak velocity of systolic (S), diastolic (D), and atrial reversal waves (Ar). TDI was used to measure annular systolic velocity (Sa), early diastolic (Ea), and late diastolic (Aa) velocity at septal and lateral walls. Pulmonary artery systolic pressure (PASP) was calculated as follows: SPAP = tricuspid transvalvular pressure difference + right atrial estimated pressure (Becker et al., 2006). The tricuspid pressure difference was calculated by the simplified Bernoulli equation: pressure difference = 4V2; the right atrial pressure was estimated by measuring the ratio of the width of the inferior vena cava and collapse on inhalation, according to the recommended method (Fleming et al., 2011). Left ventricular end-diastolic volume (EDV), end-systolic volume (ESV), and ejection fraction (LVEF) were obtained by the Simpson biplanar method.

### Analysis of 2D Strain and Torsion Parameters

Datum of speckle tracking was analyzed offline using dedicated automated software EchoPAC PC, Version 113 (GE Healthcare, Milwaukee, WI, United States). The myocardial region of interest (ROI) of the left ventricular was defined by the endocardial border. For the 2D longitudinal speckle tracking analysis, three ROIs were placed in an end-diastolic frame from the apical three-, four-, and two-chamber views, respectively. If necessary, the width of the ROI can be adjusted manually to exclude the pericardium.

Peak systolic longitudinal strain (LS) was analyzed from apical views in all 17-left ventricular (LV) longitudinal segments and averaged to LV global longitudinal strain. Longitudinal strains of the right ventricle (RV) in all six segments of the right ventricular free wall and septum or three segments of right ventricular free wall were analyzed from the apical four-chamber view and were averaged into RV global longitudinal strain. Circumferential strain (CS) and radial strain (RS) were assessed in the parasternal short-axis views at the level of the mitral valve, papillary muscle, and apical view, and the left ventricular torsion and torque were calculated.

### Statistical Analysis

The values of left and right ventricular LS and left ventricular torsion were transformed into z-scores normalized to body surface area and adjusted for heteroscedasticity presented by Dallaire et al. (2016). Z-scores for the rest of the parameters was done using the cardio z application for pediatrics (Chubb and Simpson, 2012). All datum were analyzed using the statistical package SPSS 19.0 (Chicago, IL, United States). For normally distributed continuous variables, a *t*-test was conducted to compare differences in means. Likewise, the log-rank test was used for comparing variables with non-normal distribution. Data are presented as mean and *SD* or median (interquartile range). *P* < 0.05 was considered significant.

## Results

### Clinical Characteristics and Conventional Ultrasound Analyses

There were no significant differences in age, gestational week of birth, or parental characteristics (height, weight, age, body mass index) between the children in the severe PE group and the control group. Children delivered from severe PE presented less weight (24.41 vs. 20.89 kg, *P* < 0.05), shorter height (124.1 vs. 115.6 cm, *P* < 0.05) and faster heart rates (84 vs. 93 bpm, *P* < 0.05). There were no differences in other conventional echocardiography values (*P* > 0.05, Table 1).

**TABLE 1:** Demographic characteristics and conventional echocardiography values of the study groups.

	Controls *n* = 23	Severe preeclampsia *n* = 23	*P*
Gestational age (w)	33.39 ± 2.35	34.48 ± 1.65	0.076
Birth weight (kg)	2.2 ± 0.58	1.9 ± 0.48	0.077
Age (Y)	6 ± 0.99	6 ± 0.78	0.254
Height (cm)	124.1 ± 8.40	*115.6 ± 6.54*	0.000*
Weight (kg)	*24.41 ± 4.45*	*20.89 ± 3.01*	0.004*
Body mass index (kg/m^2^)	*19.57 ± 2.60*	*18.04 ± 1.99*	0.031*
Body surface area (m^2^)	0.92 ± 0.11	0.81 ± 0.08	0.007*
Systolic blood pressure (mmHg)	88 ± 7.32	91 ± 8.21	0.218
Systolic blood pressure z score	−0.98 ± 0.70	−0.38 ± 0.76	0.397
Diastolic blood pressure (mmHg)	55 ± 12.19	55 ± 10.61	0.868
Diastolic blood pressure z score	−0.26 ± 1.14	−0.03 ± 0.92	0.375
Heart rate (bpm)	*84 ± 5.44*	*93 ± 3.54*	0.000*
Aortic root Inner diameter (mm)	20.3 ± 1.96	19.3 ± 1.94	0.089
Aortic root Inner diameter z score	−0.06 ± 0.69	−0.05 ± 0.68	0.988
Left atrium volume index (ml/m^2^)	18.61 ± 5.43	16.43 ± 4.36	0.142
Left ventricular septal diastolic thickness (mm)	6.17 ± 0.65	5.87 ± 0.63	0.113
Left ventricular septal diastolic thickness z score	0.19 ± 0.46	0.22 ± 0.42	0.829
Left ventricular posterior wall diastolic thickness (mm)	6.00 ± 0.60	5.70 ± 0.64	0.103
Left ventricular posterior wall diastolic thickness z score	0.70 ± 0.41	0.68 ± 0.53	0.881
Left ventricular mass index (g/m^2^)	65.18 ± 9.74	61.43 ± 13.83	0.294
standardized left ventricular end-diastolic volume (ml/m^2^)	40.52 ± 6.24	42.09 ± 7.20	0.435
Standardized left ventricular end-systolic volume (ml/m^2^)	14.91 ± 1.70	15.09 ± 2.97	0.809

“*” and “ltalicized” indicates p < 0.05, statistically significant compared to the control group.

### Left Ventricular Systolic and Diastolic Function

The left ventricular systolic and diastolic function of children in the study group is similar to that of children in the control group (*P* > 0.05, Table 2).

**TABLE 2:** Left ventricular (LV) function of the severe preeclampsia (PE) group and the controls.

	Controls *n* = 23	Severe preeclampsia *n* = 23	*P*
**Left ventricular systolic function**			
Left stroke volume (ml)	23.61 ± 6.17	21.91 ± 5.44	0.328
Left cardiac output (L/min)	1.97 ± 0.48	2.05 ± 0.53	0.630
Left ejection fraction (%)	62.70 ± 4.65	63.52 ± 4.10	0.526
Left ventricular myocardial performance index (LV MPI)	0.40 ± 0.05	0.41 ± 0.06	0.367
LV MPI z score	1.30 ± 0.73	1.13 ± 0.70	0.418
**Left ventricular diastolic function**			
Mitral E wave (cm/s)	104.6 ± 18.52	104.9 ± 13.43	0.949
Mitral E wave z score	0.69 ± 1.25	0.71 ± 0.91	0.950
Mitral A wave (cm/s)	53.4 ± 13.37	53.3 ± 12.95	0.982
Mitral A wave z score	0.17 ± 0.84	0.31 ± 1.04	0.628
Mitral E/A ratio	2.03 ± 0.33	2.10 ± 0.71	0.692
Mitral E/A ratio z score	0.03 ± 0.69	−0.03 ± 0.81	0.770
Mitral e’ (cm/s)	16.2 ± 1.52	15.2 ± 2.37	0.089
Mitral e’ z score	−0.24 ± 0.42	−0.53 ± 0.64	0.073
Left ventricular E/e’ (cm/s)	6.52 ± 1.44	7.08 ± 1.57	0.213
Left ventricular E/e’ z score	0.38 ± 0.76	0.67 ± 0.82	0.212
Mitral E deceleration time (ms)	158 ± 28.37	150 ± 31.13	0.355
Tricuspid valve pressure difference (mmHg)	19 ± 7.87	20 ± 7.14	0.521
Peak systolic pulmonary vein velocity (S wave, cm/s)	55.9 ± 7.62	53.6 ± 7.90	0.360
Peak pulmonary vein flow velocity in early diastole (D wave, cm/s)	65.3 ± 8.11	61.5 ± 7.87	0.146
Peak pulmonary vein flow velocity during atrial systole (Ar wave, cm/s)	28.0 ± 3.13	28.6 ± 4.19	0.621
Pulmonary vein A wave duration (ms)	82 ± 15.89	83 ± 15.31	0.803

### Right Ventricular Systolic and Diastolic Function

We also did not find significant differences in right ventricular systolic and diastolic function between the children in the experimental group and the control group (*P* > 0.05, Table 3).

**TABLE 3:** Right ventricular (RV) systolic and diastolic function of the study groups.

	Control *n* = 23	Severe preeclampsia *n* = 23	*P*
**Right ventricular systolic function**			
Right ventricular fractional area change (rvfac)	0.47 ± 0.06	0.47 ± 0.09	0.985
Right ventricular myocardial performance index (RV MPI)	0.48 ± 0.08	0.47 ± 0.09	0.693
RV MPI z score	1.30 ± 0.73	1.20 ± 0.78	0.655
Tricuspid annulus lateral wall S’ (cm/s)	12.3 ± 1.56	12.0 ± 1.24	0.391
Tricuspid annulus lateral wall S’ z score	−0.52 ± 0.78	−0.70 ± 0.62	0.391
Tricuspid annulus displacement (mm)	18 ± 1.93	17 ± 1.70	0.175
**Right ventricular diastolic function**			
Tricuspid E wave (cm/s)	69.5 ± 8.79	71.3 ± 13.77	0.616
Tricuspid E wave z score	0.70 ± 0.57	0.78 ± 0.99	0.758
Tricuspid A wave (cm/s)	41.3 ± 10.47	44.8 ± 8.98	0.256
Tricuspid A wave z score	0.05 ± 0.97	0.22 ± 0.83	0.534
Tricuspid E deceleration time (ms)	164 ± 37.55	149 ± 28.30	0.165
Tricuspid E/A ratio	1.74 ± 0.28	1.59 ± 0.35	0.160
Tricuspid E/A ratio z score	0.62 ± 0.69	0.27 ± 0.89	0.173
Tricuspid e’ (cm/s)	15.6 ± 2.09	15.9 ± 2.28	0.672
Tricuspid e’ z score	−0.29 ± 0.70	−0.21 ± 0.76	0.723
Right ventricular E/e’	4.52 ± 0.97	4.55 ± 1.19	0.927
Right ventricular E/e’ z score	0.86 ± 0.87	0.83 ± 0.87	0.927
Right atrial volume index (ml/m^2^)	15.94 ± 3.84	15.61 ± 4.99	0.800

### Myocardial Strain and Torsion

We found no significant differences in global left ventricular longitudinal strain, radial strain, circumferential strain, and right ventricular longitudinal strain between the children in the study groups (*P* > 0.05, Table 4).

**TABLE 4:** Comparison of myocardial strain and torsion of the study groups.

	Control	Severe preeclampsia	*P*
Left ventricular longitudinal strain,%	−25.96 ± 2.63	−25.74 ± 2.48	0.766
Left ventricular circumferential strain,%	−22.60 (−21.70, −24.90)	−20.90 (−19.30, −24.20)	0.223
Left ventricular radial strain,%	45.70 (35.30, 50.50)	38.10 (35.30, 49.60)	0.364
Left ventricular torsion (degree)	14.00 (11.70, 19.40)	13.30 (10.60, 15.40)	0.358
Left ventricular torsion z score	1.69 (0.91, 3.67)	1.49 (0.55, 2.26)	0.374
Right ventricular longitudinal strain,%	−27.37 ± 2.61	−28.57 ± 3.57	0.201

## Discussion

To our knowledge, this study demonstrated for the first time that the myocardial function measured by 2D speckle-tracking echocardiography in preterm children aged 5–8 years of pregnancy complicated by severe preeclampsia was not organically impaired macroscopically. However, children delivered from severe PE presented less weight (24.41 vs. 20.89 kg, *P* < 0.05), shorter height (124.1 vs. 115.6 cm, *P* < 0.05) and faster heart rates (84 vs. 93 bpm, *P* < 0.05). We believed that growth retardation of premature children is induced by placental insufficiency secondary to severe preeclampsia. The results of this study are in line with a previous study in male children exposed to severe preeclampsia, suggesting 1 kg lighter and 3 cm shorter than normal children at 2 years old and a.1 kg/m^2^ lower body mass index at 12 years old (Byberg et al., 2017). A systematic review of observational studies confirmed a significant inverse correlation between heart rate and age (Fleming et al., 2011). They found the median heart rate gradually declined from one month of age to 18 years of age. Thus, the preterm children exposed to severe PE lagged behind their peers in growth and development after birth, resulting in a relatively slow decline in their heart rate.

Heart rate is one of the most important indicators of myocardial oxygen consumption. It is suggested that resting heart rate is also an important marker of outcomes in CVD (Böhm et al., 2015). An increase in heart rate implies an increase in energy demand and myocardial oxygen demand. Similar to our results, Drude et al. observed a faster heart rate in children born to preeclampsia (Fugelseth et al., 2011). Increased heart rate may increase myocardial ischemia and electrical instability, and cardiovascular disease risk in the long run (den Hoed et al., 2013). However, the relationship between children’s heart rate and their intrauterine environment during the fetal period remains to be explored.

Myocardial strain is an important indicator reflecting myocardial deformation and the latter is a sensitive marker of myocardial dysfunction (Chen et al., 2019). The myocardial movement generated by left ventricular longitudinal contraction and diastole plays a major role in left ventricular ejection (Thapa et al., 2014). Particularly, the changes in the longitudinal strain of the subendocardial myocardium can reflect myocardial lesions more sensitively (Stanton et al., 2009). The 2D speckle tracking echocardiography (2D STE) technique shows satisfactory reproducibility when used to measure ventricular longitudinal and circumferential strains (Amundsen et al., 2006). Compared with cardiac magnetic resonance imaging, the sensitivity and specificity of the 2D-STE technique are better to accurately identify the segments with normal and abnormal motion (Becker et al., 2006). Our study could not demonstrate significant impairment in a myocardial strain of children delivered from severe PE, which suggests that there is no organic structural and functional impairment of the heart considering clinical cardiac function.

The Myocardial Performance Index (MPI) is a novel index that can be used to evaluate the cardiac function of various heart diseases comprehensively and is independent of the geometry of the ventricles, the quality of 2D images, blood pressure, and heart rate. The higher the MPI value, the worse the heart function. Although some studies reported higher MPI values in the offspring exposed to the intrauterine environment of severe PE compared to non-exposed during the fetal period (Bhorat et al., 2017) and within 48 h after birth (Mutlu et al., 2018), it is unknown whether the impairment of cardiac function persists to childhood. In our study, this indicator still did not differ between the two groups.

## Limitations

This study has some strengths and limitations worth commenting on. There were no differences in the age, gestational week of birth, or parental characteristics (height, weight, age, body mass index) between the children in the experimental group and the control group. Pregnancies with diseases that may affect the development of fetal heart, such as gestational diabetes mellitus, maternal hematologic diseases, immune system diseases, malignant tumors, heart disease, intrauterine infections, fetal congenital heart disease, or other deformities, are excluded to minimize the impact of confounding factors. But we acknowledge that PE could increase the incidence of congenital heart disease, fetal malformations, and even death. Our exclusion made us inclined to select patients less affected by preeclampsia for this research. In addition, our study was conducted on preterm children from only one medical center which might impact the variables analyzed.

## Conclusion

In conclusion, this study demonstrated that exposure to severe preeclampsia during pregnancy did not have a significant impact on the clinical cardiac systolic and diastolic function of offspring at 5–8 years old. However, their faster resting heart rate may have suggested a negative impact on the heart and could be a potential indicator of an increased risk of cardiovascular disease in the future.

## Data Availability Statement

The original contributions presented in the study are included in the article/supplementary material, further inquiries can be directed to the corresponding author/s.

## Ethics Statement

The studies involving human participants were reviewed and approved by Ethics Committee of The First Affiliated Hospital of Soochow University. Written informed consent was obtained from the individual(s), and minor(s)’ legal guardian/next of kin, for the publication of any potentially identifiable images or data included in this article.

## Author Contributions

BH and BZ: conceptualization. HC, YG, and FS: data curation and writing—original draft preparation. BZ: echocardiographic image acquisition. BH, JF, and XG: supervision. All authors have read and agreed to the published version of the manuscript.

## Conflict of Interest

The authors declare that the research was conducted in the absence of any commercial or financial relationships that could be construed as a potential conflict of interest.

## Publisher’s Note

All claims expressed in this article are solely those of the authors and do not necessarily represent those of their affiliated organizations, or those of the publisher, the editors and the reviewers. Any product that may be evaluated in this article, or claim that may be made by its manufacturer, is not guaranteed or endorsed by the publisher.

## References

[B1] Amundsen B. H. Helle-Valle T. Edvardsen T. Torp H. Crosby J. Lyseggen E. (2006). Noninvasive myocardial strain measurement by speckle tracking echocardiography: validation against sonomicrometry and tagged magnetic resonance imaging. *J. Am. Coll. Cardiol.* 47 789–793. 10.1016/j.jacc.2005.10.040 16487846

[B2] Arnott C. Skilton M. R. Ruohonen S. Juonala M. Viikari J. S. Kähönen M. (2015). Subtle increases in heart size persist into adulthood in growth restricted babies: the Cardiovascular Risk in Young Finns Study. *Open Heart* 2:e000265. 10.1136/openhrt-2015-000265 26339495 PMC4555072

[B3] Becker M. Bilke E. Kühl H. Katoh M. Kramann R. Franke A. (2006). Analysis of myocardial deformation based on pixel tracking in two dimensional echocardiographic images enables quantitative assessment of regional left ventricular function. *Heart* 92 1102–1108. 10.1136/hrt.2005.077107 16387826 PMC1861114

[B4] Bhorat I. E. Bagratee J. S. Reddy T. (2017). Assessment of fetal myocardial performance in severe early onset pre-eclampsia (EO-PET) with and without intrauterine growth restriction across deteriorating stages of placental vascular resistance and links to adverse outcomes. *Eur. J. Obstet. Gynecol. Reprod. Biol.* 210 325–333. 10.1016/j.ejogrb.2017.01.014 28113071

[B5] Böhm M. Reil J. C. Deedwania P. Kim J. B. Borer J. S. (2015). Resting heart rate: risk indicator and emerging risk factor in cardiovascular disease. *Am. J. Med.* 128 219–228. 10.1016/j.amjmed.2014.09.016 25447617

[B6] Byberg K. K. Øymar K. Eide G. E. Forman M. R. Júlíusson P. B. (2017). Exposure to preeclampsia in utero affects growth from birth to late childhood dependent on child’s sex and severity of exposure: follow-up of a nested case-control study. *PLoS One* 12:e0176627. 10.1371/journal.pone.0176627 28486480 PMC5423584

[B7] Chen X. Li L. Cheng H. Song Y. Ji K. Chen L. (2019). Early Left Ventricular Involvement Detected by Cardiovascular Magnetic Resonance Feature Tracking in Arrhythmogenic Right Ventricular Cardiomyopathy: the Effects of Left Ventricular Late Gadolinium Enhancement and Right Ventricular Dysfunction. *J. Am. Heart Assoc.* 8:e012989. 10.1161/JAHA.119.012989 31441357 PMC6755833

[B8] Cheng S. B. Sharma S. (2016). Preeclampsia and health risks later in life: an immunological link. *Semin. Immunopathol.* 38 699–708. 10.1007/s00281-016-0579-8 27339196

[B9] Chubb H. Simpson J. M. (2012). The use of Z-scores in paediatric cardiology. *Ann. Pediatr. Cardiol.* 5 179–184. 10.4103/0974-2069.99622 23129909 PMC3487208

[B10] Dallaire F. Slorach C. Bradley T. Hui W. Sarkola T. Friedberg M. K. (2016). Pediatric Reference Values and Z Score Equations for Left Ventricular Systolic Strain Measured by Two-Dimensional Speckle-Tracking Echocardiography. *J. Am. Soc. Echocardiogr.* 29 786–793.e8. 10.1016/j.echo.2016.03.018 27185223

[B11] Davis E. F. Lewandowski A. J. Aye C. Williamson W. Boardman H. Huang R. C. (2015). Clinical cardiovascular risk during young adulthood in offspring of hypertensive pregnancies: insights from a 20-year prospective follow-up birth cohort. *BMJ Open* 5:e008136. 10.1136/bmjopen-2015-008136 26105032 PMC4480003

[B12] den Hoed M. Eijgelsheim M. Esko T. Brundel B. J. Peal D. S. Evans D. M. (2013). Identification of heart rate-associated loci and their effects on cardiac conduction and rhythm disorders. *Nat. Genet.* 45 621–631. 10.1038/ng.2610 23583979 PMC3696959

[B13] Fleming S. Thompson M. Stevens R. Heneghan C. Plüddemann A. Maconochie I. (2011). Normal ranges of heart rate and respiratory rate in children from birth to 18 years of age: a systematic review of observational studies. *Lancet* 377 1011–1018. 10.1016/S0140-6736(10)62226-X 21411136 PMC3789232

[B14] Fugelseth D. Ramstad H. B. Kvehaugen A. S. Nestaas E. Støylen A. Staff A. C. (2011). Myocardial function in offspring 5-8years after pregnancy complicated by preeclampsia. *Early Hum. Dev.* 87 531–535. 10.1016/j.earlhumdev.2011.04.006 21550734

[B15] Jonker S. S. Giraud M. K. Giraud G. D. Chattergoon N. N. Louey S. Davis L. E. (2010). Cardiomyocyte enlargement, proliferation and maturation during chronic fetal anaemia in sheep. *Exp. Physiol.* 95 131–139. 10.1113/expphysiol.2009.049379 19700519 PMC2906234

[B16] Mutlu K. Karadas U. Yozgat Y. Meşe T. Demirol M. Coban S. (2018). Echocardiographic evaluation of cardiac functions in newborns of mildly preeclamptic pregnant women within postnatal 24-48 hours. *J. Obstet. Gynaecol.* 38 16–21. 10.1080/01443615.2017.1322564 28631496

[B17] Peixoto A. B. Rolo L. C. Nardozza L. Araujo Júnior E. (2018). Epigenetics and Preeclampsia: programming of Future Outcomes. *Methods Mol. Biol.* 1710 73–83. 10.1007/978-1-4939-7498-6_6 29196995

[B18] Stanton T. Ingul C. B. Hare J. L. Leano R. Marwick T. H. (2009). Interaction of left ventricular geometry and myocardial ischemia in the response of myocardial deformation to stress. *Am. J. Cardiol.* 104 897–903. 10.1016/j.amjcard.2009.05.028 19766753

[B19] Thapa P. Xing Y. Y. Li Y. H. (2014). Mitral annulus displacement measured by two-dimensional speckle tracking imaging to assess the left ventricular longitudinal systolic function in coronary heart disease. *J. Clin. Ultrasound* 42 544–549. 10.1002/jcu.22181 24942839

[B20] Zhang L. (2005). Prenatal hypoxia and cardiac programming. *J. Soc. Gynecol. Invest.* 12 2–13. 10.1016/j.jsgi.2004.09.004 15629664

